# The State of HIV‐Related Implementation Research in 2026: Lessons Learned and Future Directions

**DOI:** 10.1002/jia2.70213

**Published:** 2026-10-08

**Authors:** Stefan Baral, Tonderai Mabuto, Sheree Schwartz

**Affiliations:** ^1^ Johns Hopkins Implementation Science Hub Department of Epidemiology Johns Hopkins School of Public Health Baltimore Maryland USA; ^2^ Department of Implementation Research The Aurum Institute Johannesburg South Africa

## 1 Introduction

The quality and quantity of HIV‐related implementation research have continued to accelerate over the past 15 years [1, 2, 3]. As a field, we have dramatically increased funding opportunities related to implementation science, while simultaneously increasing academic productivity [2]. During recent years, the rigour of implementation science‐related work has also increased, with expectations of use of implementation research theories, models and frameworks, as well as increased reporting of implementation outcomes [2]. Despite these successes, there is also recognition of areas for progress [4]. Reporting implementation outcomes beyond acceptability, appropriateness and feasibility is a critical area for expansion as the field moves down the translational pathway beyond pre and/or early implementation work, to increasing focus on adoption, fidelity and maintenance [5]. Unpacking mechanisms and linking implementation outcomes more directly to health outcomes is similarly in need of increased attention. Increased specificity of implementation strategies and study of those strategies through research focused on enhancing implementation outcomes, such as through hybrid type 2 and 3 effectiveness‐implementation studies, represent other growth opportunities for HIV‐related implementation research. Further, including organizational readiness, culture and other inner setting service delivery level features as targets for implementation strategies and change, alongside consideration of outer setting context such as varying healthcare financing models or policy contexts are critical areas for analysis [6].

This special issue seeks to advance science through addressing gaps in the HIV‐related implementation research space. The collection of articles spanning multiple regions around the world, a number of which are multi‐site studies, provides best practice examples of moving beyond barriers and facilitators to advancing implementation. Together, this work demonstrates opportunities to impact health at scale through increased focus on the behaviour of the health ecosystems and not just the individual patients or clients within those systems. Multiple cross‐cutting themes emerge across the collection, including the importance of specifying implementation strategies, defining the mechanisms through which those strategies shape real‐world impact and measuring implementation outcomes as predictors of programme effectiveness. The papers also underscore the central role of community engagement and culturally grounded approaches in addressing structural determinants of HIV prevention and care. Finally, the issue highlights HIV‐related implementation research as a growing but still underdeveloped funding priority, with continued opportunities to expand the methodological rigour, scale and policy relevance of the field.

## 2 Lessons Learned

### 2.1 The Benefits of Specifying Implementation Strategies

A key theme across multiple papers centred on the development, specification and evaluation of implementation strategies in clinical programmes. Several lessons emerged from specific papers, including the importance of precision in intervention and strategy specification and measurement and the value of describing and even studying the mechanisms by which these strategies facilitate outcomes. A multi‐site study across the United States by **Meyers et al**. on injectable antiretroviral therapy programmes specified seven implementation strategies and used causal pathway diagrams to link strategies to mechanisms, moderators, and outcomes related to access and equity; most participating clinics achieved partial or full progress on access‐related outcomes by mid‐implementation [7]. Similarly, **Sales et al**. evaluated an intervention in Title X‐funded safety‐net family planning clinics providing affordable reproductive and preventive health services for underserved communities in Atlanta, United States (US) [8]. Specifically, the intervention leveraged tailored trainings, technical assistance and resources guided by the interactive systems framework, demonstrating increased clinic readiness, improved HIV testing and expansion of pre‐exposure prophylaxis (PrEP) services following implementation strategy engagement [9]. A human‐centred intervention described by **Mahone et al**. (“PrEP4Her”) employed multi‐level strategies including provider training, electronic health record tools and patient‐facing materials, demonstrating increased provider–patient discussions and modest increases in PrEP prescriptions, with Reach, Effectiveness, Adoption, Implementation and Maintenance or RE‐AIM framework used to evaluate reach and effectiveness [10]. Collectively, these papers highlight how structured specification of implementation strategies, leveraging established implementation frameworks, and tailoring to clinical workflows facilitated the uptake of complex HIV innovations.

### 2.2 Defining Implementation Strategies Improves Real‐World Impact of HIV Prevention and Treatment Programmes

A second key theme across the special issue was the use of implementation strategies focused on expanding HIV prevention and care and improving quality and the efficiency of service delivery. These studies further highlight the centrality of mixed methods research to implementation research and the utility of determinants frameworks in structuring assessments of barriers and facilitators to evidence‐based interventions. A mixed‐methods evaluation by **Golub et al**. of a health‐department‐driven quality‐based financing model in New York City, US demonstrated that defining programme quality through implementation strategies — universal screening, holistic care and sex‐positive counselling — was acceptable, feasible, and associated with improved service integration and reduced stigma [11]. Similarly, a multi‐country synthesis by **Aifah et al**. of six trials completed in settings across sub‐Saharan Africa integrating hypertension care into HIV services identified 10 cross‐cutting implementation strategies, with healthcare worker training, audit and feedback, and practice facilitation consistently used across settings, though adapted to local contexts in delivery and intensity [12]. Finally, **Shaw et al**. characterized strategies supporting integrated services across community‐based organizations in King County, Washington, US, identifying a large set of stakeholder‐generated strategies emphasizing organizational infrastructure, training, communication and community engagement, and highlighting feasibility–impact trade‐offs across implementation contexts [13]. Taken together, these articles highlight the utility of specifying implementation strategies of well‐established interventions including PrEP and antiretroviral therapy (ART) as a means of optimizing the real‐world HIV prevention and treatment impacts.

### 2.3 The Importance of Measuring Implementation Outcomes as Predictors of Programme Effectiveness

A number of studies reinforced the utility of implementation outcomes as intermediate outcomes between the effectiveness of the implementation strategy and the real‐world impact of the underlying intervention. A range of implementation outcomes represented the focus of studies, including fidelity, adaptation, and quality of implementation across diverse domestic US and international settings. A stepped‐wedge cluster randomized trial by **Mukui et al**. assessed fidelity to PrEP delivery in family planning clinics in Kenya and showed generally high engagement with key service components but variable completeness of counselling, promotion and safety assessments, underscoring the need for ongoing quality improvement and provider support [14]. In Malawi, **Tembo et al**. led a cluster randomized trial demonstrating that a decentralized digital training strategy significantly improved healthcare worker fidelity to index case testing protocols compared to standard training [15]. A school‐based intervention in the rural uMkhanyakude District of South Africa described by **Chimbindi et al**. detailed a participatory co‐adaptation process for a multi‐component sexual and reproductive health programme, showing that adaptation to local context enabled feasible delivery in the context of persistent structural barriers in schools, including resource and infrastructure constraints [16]. Specifically, Chimbindi et al. leveraged qualitative methods to underscore how implementation outcomes are shaped by organizational structures and delivery systems and how innovations in service delivery models and workflow innovations can improve HIV care. **Zhao et al**. used comparative workflow modelling to examine status‐neutral HIV care implementation across six high‐burden US [17] jurisdictions, identifying variation in workflow design, staff roles, service integration and timing of activities as key determinants of implementation approaches. Taken together, these studies demonstrate a range of measurement approaches for implementation outcomes and the potential for the improvement of implementation outcomes as an important target of study. Specifically, a range of interventions, including training, co‐adaptation and monitoring, can be used to improve implementation outcomes, which represents the improved delivery of evidence‐based interventions.

### 2.4 The Fundamental Role of Community Engagement in HIV‐Related Implementation Research

Building on the study of implementation strategies and outcomes, there were also contributions focused on community‐engaged and structural approaches to HIV prevention and care. Specifically, these studies highlighted the interventions targeting measurable structural determinants of engagement in HIV prevention and treatment services as well as stigma. A multi‐site study in cities across the United States, including San Juan, Puerto Rico; New York City, New York; Philadelphia, Pennsylvania by **Martinez et al**. examined medical‐legal partnerships that embedded legal services within healthcare settings to address legal needs, demonstrating strong feasibility, acceptability and improved engagement in HIV care using RE‐AIM and the Consolidated Framework for Implementation Research frameworks [18]. A barbershop‐based intervention in Tennessee, US, described by **Perkins et al.,** trained barbers as health ambassadors and used education, referrals, and social media to mitigate HIV‐related stigma and promote HIV prevention services [19]. Implementation outcomes in this study were consistently high across feasibility, acceptability and appropriateness, with associated increases in HIV knowledge and service referrals. Another community‐partnered project by **Wilson et al**. (“Acción PrEP”) used participatory design in Mecklenburg County, North Carolina, US to co‐develop implementation strategies for Latine populations, demonstrating high acceptability and feasibility of strategies including needs assessments, blended facilitation and culturally tailored campaigns, while identifying staffing and resource constraints as key implementation barriers [20]. Collectively, these studies demonstrate the value of community engagement and culturally grounded strategies in addressing structural inequities and improving intervention uptake.

### 2.5 HIV‐Related Implementation Research as a Funding Priority

Systematically assessing funding for HIV‐related implementation research is central to highlighting key priorities to realize real‐world impacts of HIV‐prevention and treatment research supported by the U.S. National Institutes of Health (NIH). To that end, **Donenberg et al**. conducted an NIH portfolio analysis of funded research from 2021 to 2024, documenting substantial growth in implementation science investment, increasing use of hybrid study designs, mixed methods approaches, and widespread application of established implementation determinants and evaluation frameworks [21]. Despite this growth, the authors note that implementation science remains a relatively small proportion of the NIH's overall HIV research portfolio, highlighting opportunities for continued expansion.

Taken together, this collection of articles advances the application of implementation science methodologies within HIV‐related research. Across diverse settings, populations and interventions, these studies reflect the increasing use of multi‐component and combination implementation strategies to address the complexity of real‐world implementation challenges. As these approaches become more common, greater attention will be needed to articulate the functions of individual strategy components, their mechanisms of action and their reciprocal interaction with the context in which they are implemented and evaluated. Such understanding will be essential for advancing cumulative learning and supporting adaptation and scale‐up [22]. Equally important is the continued evolution of implementation outcomes from measures of implementation success to explanatory constructs that serve as targets of implementation strategies, mediate downstream health outcomes and illuminate implementation processes over time.

Meaningful partnerships with implementers and end‐users will remain central to the field, requiring greater attention to co‐design processes, power dynamics and the transparency of implementation decision‐making and adaptation. As co‐creation approaches become more common, implementation research will require robust methods for assessing partnership quality, shared decision‐making and other dimensions of meaningful engagement [23, 24, 25, 26]. Similarly, while policymakers are increasingly being included as partners in participatory and implementation research, greater attention to policy implementation and policy evaluation research is needed to understand how evidence informs policy processes and shapes resource allocation and service delivery environments [27].

Sustainability considerations must increasingly be incorporated into implementation decision‐making, recognizing that implementation strategies themselves need to withstand changing funding environments, workforce constraints and shifting health system priorities [28, 29]. This is particularly urgent given substantial reductions in dedicated support for HIV prevention and treatment programmes globally, a trend that may continue and place even greater importance on designing services and implementation strategies that can be sustained, integrated and scaled within existing health systems. The studies in this special issue further highlight the growing importance of integrating HIV services across health services, sectors and delivery platforms as health systems increasingly prioritize efficiency and sustainability. Implementation researchers can support this agenda by identifying the core functions, organizational capabilities and combinations of implementation strategies that enable successful integration across diverse settings and contexts [30].

Notably, as implementation science becomes more embedded within routine service delivery, important tensions become more visible. Historically, many implementation‐oriented studies have benefited from dedicated research funding, personnel and infrastructure; increasingly, however, implementation questions will need to be answered within routinely funded government and health‐system services, often without substantial additional resources for research or implementation support [31]. Adaptation to context, responsiveness to implementation realities and the need for timely service improvements must often be balanced against requirements for rigour, reproducibility and causal inference. Similarly, tensions between fidelity and adaptation, and between effectiveness and feasibility, require deliberate and transparent navigation rather than methodological resolution.

## 3 Future Directions

Methodological innovation must keep pace with programme complexity. Adaptive, flexible and hybrid study designs, transportability, and advances in causal inference represent necessary methodological innovations given the increasing complexity of biomedical and socio‐behavioural interventions in HIV [3, 24, 31]. Moreover, implementation researchers should actively leverage advances in technology allowing for improved modelling of implementation strategies, digital twinning to support causal inference and agentic artificial intelligence. By combining routinely collected health‐system data with other large‐scale and non‐conventional data sources, these technologies may allow researchers to simulate how implementation strategies could perform under different conditions, examine why and for whom strategies work, and adapt strategy selection as contextual conditions change. These technological advances offer opportunities to interrogate mechanisms by examining the pathways through which implementation strategies influence provider behaviour, service delivery and patient outcomes, optimize strategy selection and tailoring, and support learning health systems [32]. In this context, a learning health system is one in which data and implementation research are embedded within routine service delivery and used continuously to identify gaps, test improvements and adapt services in response to emerging evidence and changing needs.

Ultimately, the body of work presented in this special issue represents significant progress in the field of HIV‐related implementation research. Moving forward necessitates the continued advance of the methods and scale of HIV‐related implementation research to achieve our collective goal of ending HIV as a public health threat.

## Author Contributions

**Stefan Baral**: conceptualization, project administration, writing – original draft, writing – review and editing. **Tonderai Mabuto**: conceptualization, writing – original draft, writing – review and editing. **Sheree Schwartz**: conceptualization, writing – original draft, writing – review and editing.

## Funding

This special issue was funded by the Johns Hopkins Implementation Science Hub (R24AI191865) and the Implementation Science Core of the Johns Hopkins Center for AIDS Research (P30AI094189). SB was supported by the Center for HIV and Mental Health Elimination Strategies (CHIMES) (P30MH136919). TM was supported by the National Institute for Health and Care Research (NIHR; grant number NIHR305519) with UK international development funding from the UK Government to support global health research.

## Disclaimer

The views expressed in this article are those of the authors and not necessarily those of the NIHR or the UK Government.

## Conflicts of Interest

The authors declare no conflicts of interest.
